# Socio-medical determinants of timely breastfeeding initiation in Ethiopia: Evidence from the 2011 nation wide Demographic and Health Survey

**DOI:** 10.1186/s13006-015-0050-9

**Published:** 2015-08-19

**Authors:** Yihunie Lakew, Lianna Tabar, Demewoz Haile

**Affiliations:** Ethiopian Public Health Association, Addis Ababa, Ethiopia; WEEMA International, Brookline, MA USA; Department of Reproductive Health, College of Medicine and Health Sciences, Bahir Dar University, Bahir Dar, Ethiopia

## Abstract

**Background:**

Early initiation of breastfeeding is a simple and cost effective intervention to advance the health of mothers and newborns. The World Health Organization (WHO) and United Nations Children’s Fund (UNICEF) recommend that breastfeeding should be initiated within one hour of birth. The aim of this study is to identify factors associated with timely initiation of breastfeeding among mothers in Ethiopia within one hour of birth.

**Methods:**

This study used data from the 2011 Ethiopia Demographic and Health Survey. A total of 11,654 households were included in the analysis from 11 administrative states of Ethiopia. Bivariate and multivariable logistic regression models with adjusted odds ratio (AOR) and 95 % confidence intervals (CI) were used to quantify predictors of early breastfeeding initiation.

**Results:**

The overall prevalence of timely breastfeeding initiation was 52 % (95 % CI: 51.09, 52.91). The prevalence was higher in urban settings (61.4 %; 95 % CI: 58.85, 63.85) than in rural areas (52.3 %; 95 % CI: 51.33, 53.28). The highest prevalence was found in Addis Ababa 71.5 % (95 % CI: 64.88, 77.12) while the lowest prevalence was 41.7 % (95 % CI: 36.62, 47.00) in Somali regional state. Multivariable logistic regression analysis showed that rural mothers had 39 % lower odds of timely breastfeeding initiation (AOR 0.61; 95 % CI: 0.50, 0.76) compared to urban mothers. Mothers who had secondary education or higher had 60 % higher odds of timely breastfeeding initiation (AOR 1.6; 95 % CI: 1.02, 2.44) than never educated mothers. Mothers who had caesarian deliveries had 61 % lower odds of timely breastfeeding initiation (AOR 0.39; 95 % CI: 0.22, 0.71) compared to vaginal deliveries. Working mothers were 23 % less likely to timely initiate breastfeeding (AOR 0.77; 95 % CI: 0.69, 0.85) compared to housewives. Female infants had a 20 % higher chance of timely breastfeeding initiation (AOR 1.2; 95 % CI: 1.05, 1.30) compared to male infants.

**Conclusion:**

Early initiation of breastfeeding within the first hour after birth was not optimal  in Ethiopia. Factors such as place of residence, educational level, occupation, gender of the newborn and type of delivery should be considered in any intervention program in order to enhance timely breastfeeding initiation.

## Background

Early initiation of breastfeeding is a simple and cost effective intervention that benefits the health of both mothers and newborns. The World Health Organization (WHO) and United Nations Children’s Fund (UNICEF) recommend that breastfeeding be initiated within one hour of birth [1, 2].

In this article, timely breastfeeding initiation is considered as breastfeeding initiated within the first hour of birth. It is during the first 30–60 minutes after birth that the sucking reflex of babies is most active and babies are more alert [1]. Timely initiation of breastfeeding stimulates the mother’s production of breast milk and ensures more intake of the highly nutritious colostrum breast milk produced during the first few days after birth [1, 2]. Timely breastfeeding initiation is also interrelated with early contact that is important for mother-to-infant relationships and has positive effects on duration of breastfeeding [2].

Breast milk as a source of nutrition is critical to protect newborns and infants against many illnesses and infectious diseases, including reducing the risk of diarrhea [3–5], respiratory infections such as pneumonia [6], meningitis [6], neonatal sepsis [6–9] and decreasing the risk of chronic diseases later in life [10]. It has been estimated that optimal breastfeeding for children has the potential to prevent 1.4 million under five deaths in the developing world annually [11]. Timely initiation of breastfeeding is essential to ensure the baby is immunized with this ‘live fluid’ to sustain life. Twenty two percent of neonatal deaths could be prevented if all infants are put to the breast within the first hour of birth [9]. Besides benefits to the infant, if initiated soon after birth, breastfeeding also helps the mother’s uterus to contract encouraging expulsion of the placenta and reducing the risk of severe bleeding and infection [12]. Other benefits include decreased risk of subsequent breast and ovarian cancers and hip fractures [13].

Ethiopia has made encouraging improvements in accessibility and provision of primary healthcare services, especially since establishment of the Health Extension Program in 2003. To date, more than 38,000 Health Extension Workers have been deployed throughout Ethiopia to promote 16 basic health intervention packages. Early initiation of breastfeeding is a key component of the nutrition package. The importance of timely initiation of breastfeeding has been further recognized by the Ethiopian government with its inclusion in the infant and young child feeding guidelines in 2004 [14]. However, in Ethiopia, only 52 % of infants start breastfeeding within one hour of birth [15].

In African countries and elsewhere in the developing world, studies have shown that factors associated with early breastfeeding initiation include occupation [16], social support during childbirth [17], perception of lacking breast milk, performing post-birth activities such as bathing and place of delivery, some traditional ethnic beliefs [18] and type of delivery [16, 19]. In Ethiopia, very limited evidence has been available to scale up and guide efforts to improve timely breastfeeding initiation. Only small scale studies in the country have identified factors as independent predictors of timely breastfeeding initiation including place of residence [20], type of delivery [21], educational status [21] and receiving postnatal counseling [20]. Therefore, this study is intended to identify socio-medical factors associated with timely initiation of breastfeeding among mothers in Ethiopia.

## Methods

### Study setting

The 2011 Ethiopian Demographic and Health Survey (EDHS) was conducted in the nine regional states of Ethiopia; namely Tigray, Afar, Amhara, Oromia, Somali, Benishangul-Gumuz, Southern Nations Nationalities and Peoples (SNNP), Gambella and Harari and two city Administrations named Addis Ababa and Dire Dawa. Ethiopia is one of the sub-Saharan countries found in the Horn of Africa with a population of 73.5 million according to the 2007 national housing and population census [22].

### Data type and study design

This study is based on cross-sectional secondary data from the 2011 EDHS. The 2011 EDHS samples were selected using a stratified, two-stage cluster sampling design. In the first stage, the enumeration areas called clusters were selected with probability proportional to the enumeration area size with independent selection in each sampling stratum. In the second stage, a fixed number of 30 households were selected for each enumeration area. All women age 15–49 who were usual residents or who slept in the selected households the night before the survey were eligible. The survey data included a women’s questionnaire to measure socio-demographic characteristics of the mothers, information on reproductive health and service use behaviours. The tool was pretested and translated into three local languages - *Amharic, Oromefa and Tigregna*. The EDHS was designed to provide population and health indicators at national and regional levels. The survey is conducted every five years. The detailed methodology is found elsewhere [15].

### Data extraction

The 2011 EDHS data were downloaded with permission from the Measure DHS website in SPSS format. After reviewing the detailed data coding, further data recoding was performed. A total of 11,654 live births in five years preceding the survey were included in the analysis. Based on the published literature, we extracted a wide-range of socio-demographic and economic variables, health service related factors and breastfeeding initiation. The chosen variables were residence, region, wealth index, education, husband education, occupation, age, birth order, parity, Provide antenatal care (ANC) attendance, place of delivery, type of delivery, birth interval, sex of child , awareness of community conversation (CC) program, family size and exposure to mass media (indexed from television (TV), newspaper and radio).

### Measurement of variables

Timely initiation of breastfeeding was measured by asking mothers to provide information regarding the time at which their index infant was put to the breast after delivery. For this analysis, occupational status was defined as non-working and working. Any professional/technical/managerial, clerical, sales and services, skilled manual, unskilled manual and agriculture classifications were classified as working. Parity defined as the number of children ever born, was categorized as 1–4, 5–9 and 10+. Wealth index was constructed using household asset data via a principal components analysis to categorize individuals into wealth quintiles (poorest, poorer, middle, rich and richest). However, wealth index was re-categorized into three groups (poor, middle and rich) to give more meaningful and practical sub-population categories for designing program interventions in the general community. The Community Conversation (CC) program is a social mobilization method implemented in Ethiopia with an objective “to generate a response to HIV/AIDS and other health issues that integrates individual and community concerns, values, and beliefs and addresses attitudes and behaviours embedded in social systems and structures”. Trained facilitators lead community meetings over a ten month period to determine a plan of action for the community [19]. Awareness of the CC program was measured by asking women whether or not they had heard of the CC program.

### Statistical analysis

For all analysis, sample weights were applied in order to compensate for the unequal probability of selection between the strata defined by geographic location as well as for non-responses. A detailed explanation of the weighting procedure can be found in the EDHS methodology report [15]. We used “svy” in STATA version 11 to weight the survey data and do the analyses.

Descriptive statistics were used to show the prevalence of timely breastfeeding initiation with background characteristics. Bivariate and multivariable logistic regression statistical analysis was carried out to determine the factors associated with timely breastfeeding initiation. Variables found statistically significant at *p*-value < 0.25 during bivariate analysis were then included in the multivariable logistic regression model [23]. This p-value cutoff point prevented removing variables that would potentially have an effect during multivariable analysis. Both crude and adjusted odds ratios (COR and AOR) were reported with a 95 % confidence intervals (CI). Variables at *p*-value < 0.05 were considered statistically significant in the multivariable logistic regression model.

### Ethical issues

The data were downloaded and used after the purpose of the analysis was communicated and approved by Measure DHS. The original DHS data were collected in confirmation with international and national ethical guidelines. Ethical clearance for the original survey was provided by Ethiopian Public Health Institute (EPHI) Review Board, the National Research Ethics Review Committee (NRERC) at the Ministry of Science and Technology, the Institutional Review Board of ICF International, and the Center for Disease Prevention and Control (CDC).

## Results

The overall prevalence of timely initiation of breastfeeding was 52 % (95 % CI: 51.09, 52.91). The prevalence of timely breastfeeding initiation in urban setting was 61.4 % (95 % CI: 58.85, 63.85) and 52.3 % (95 % CI: 51.33, 53.28) in rural areas. Among those mothers who delivered by caesarian section, about 45.6 % (95 % CI: 37.54, 53.67) of mothers practiced timely breastfeeding initiation. The highest prevalence of timely initiation of breastfeeding was found in Addis Ababa at 71.5 % (95 % CI: 64.88, 77.12) followed by Southern Nation, Nationalities and Peoples (SNNP) regional state with 69.5 % (95 % CI: 67.66, 71.32). The lowest prevalence was 41.7 % (95 % CI: 36.62, 47.00) in Somali region. Mothers who have first birth order and were aware of community conversation program had less than 50 % prevalence of timely breastfeeding initiation. The prevalence of timely breastfeeding initiation was 61.7 % (95 % CI: 57.09, 66.38) among mothers who have secondary and higher educational status. The prevalence of timely breastfeeding initiation in all categories of wealth index, sex of child, ANC attendance, parity, occupation, birth interval, exposure to mass media and family size were almost fifty percent or more (Table 1).

**Table 1:** Timely initiation of breastfeeding by background characteristics among Ethiopian mothers, 2011

Background characteristics	Weighted total number of respondents	Prevalence of early breastfeeding initiation (95 % CI)
Wealth index		
Poor	5,225	52.2 (50.85, 53.56)
Middle	2,379	52.7 (50.70, 54.71)
Rich	3,948	53.5 (51.94, 55.05)
Residence		
Urban	1,455	61.4 (58.85, 63.85)
Rural	10,097	52.3 (51.33, 53.28)
Education		
No education	8,023	52.9 (51.8, 53.99)
Primary	3,111	53.9 (52.15, 55.65)
Secondary and higher	419	61.7 (57.09, 66.38)
Religion		
Christian	7,185	53.4 (52.25, 54.56)
Muslim	4,093	53 (51.46, 54.52)
Others	273	64 (58.28, 69.63)
Type of delivery		
Vaginal	11,407	53.6 (52.68, 54.51)
Caesarian	145	45.6 (37.54, 53.67)
Sex of child		
Male	5,984	52.2 (50.94, 53.47)
Female	5,568	54.9 (53.59, 56.21)
Occupation		
Housewife	5,346	57.6 (56.27, 58.91)
Working	6,206	53.5 (48.66, 51.15)
Antenatal clinic attendance		
Less than 4 times	6,216	51.7 (50.46, 52.95)
4 and more times	1,451	55.7 (53.12, 58.23)
Region		
Tigray	732	46.5 (42.85, 50.07)
Afar	118	64.2 (55.46, 72.65)
Amhara	2,586	41.9 (40.03, 43.83)
Oromiya	4,896	52.4 (51.01, 53.81)
Somali	345	41.7 (36.62, 47.00)
Benishangul-Gumuz	135	45.9 (37.65, 54.37)
SNNP	2,427	69.5 (67.66, 71.32)
Gambella	39	63.5 (48.27, 77.91)
Harari	28	69.4 (49.14, 83.05)
Addis Ababa	209	71.5 (64.88, 77.12)
Dire Dawa	38	67.9 (52.5, 81.63)
Aware of community conversation program		
No	8,681	54.9 (53.85, 55.95)
Yes	2,869	49.2 (47.39, 51.05)
Birth order		
1	2,195	49.5 (47.43, 51.61)
2	1,966	54.2 (52.01, 56.42)
3	1,647	53.8 (51.38, 56.2)
4	5,744	54.6 (53.31, 55.88)
Parity		
1–4	6,739	52.2 (51.01, 53.39)
5–9	4,313	55.2 (53.72, 56.69)
10+	500	56.1 (51.82, 60.51)
Place of delivery		
Home	10,368	53.1 (52.13,54.06)
Health facility	1,122	57.1 (54.31,60.09)
Birth interval		
Less than 2 years	1,904	55.7 (53.49, 57.95)
2 years and plus	7,442	54.1 (52.96, 55.23)
Family size		
1–4	2,847	51.7 (49.87, 53.54)
5–9	7,907	54.2 (53.11, 55.30)
10+	798	52.2 (48.79, 55.71)
Exposure to mass media		
No	4,754	52.8 (51.38, 54.21)
Yes	6,772	54 (52.81, 55.19)
Total	11,552	52.0 (51.09, 52.91)

Multivariable logistic regression analysis indicated that rural mothers had 39 % lower odds of timely breastfeeding initiation (AOR 0.61; 95 % CI: 0.50, 0.76) compared to urban mothers. Those mothers who had secondary and higher education level were 60 % more likely to timely initiate breastfeeding (AOR 1.6; 95 % CI: 1.02,2.44). Compared to mothers residing in Addis Ababa, mothers residing in regions of Oromiya had 53 % (AOR 0.47; 95 % CI: 0.27, 0.81), in Somali 73 % (AOR 0.27; 95%CI: 0.14, 0.50), in Benishangul-Gumuz 69 % (AOR 0.31; 95 % CI: 0.15,0.65) and in Tigray 55 % (AOR 0.45; 95 % CI: 0.25, 0.80) lower odds of timely breastfeeding initiation. Working mothers had 23 % less likely to timely initiate breastfeeding (AOR 0.77; 95 % CI: 0.69, 0.85) as compared to housewife mothers. Type of delivery was significantly associated with timely initiation of breastfeeding. Those mothers who delivered the index infant by caesarian section had 61 % lower odds of initiating breastfeeding timely (AOR 0.39; 95 % CI: 0.22, 0.71) as compared to those with vaginal delivery. Sex of the newborn was also associated with timely initiation of breastfeeding. Female newborns had a 20 % higher odds of initiating breastfeeding timely (AOR 1.2; 95 % CI: 1.05, 1.30) as compared to male newborns (Table 2).

**Table 2:** Factors associated with breastfeeding initiation within one hour among Ethiopian mothers using logistic regression model, 2011

Variables	COR (95%CI)	AOR (95%CI)
Residence		
Urban	1.00	1.00
Rural	0.69 (0.62,0.77)	0.61 (0.50,0.76)
Region		
Addis Ababa	1.00	1.00
Afar	0.72 (0.44, 1.16)	0.65 (0.31, 1.39)
Amhara	0.29 (0.21, 0.39)	0.31 (0.18, 0.55)
Oromiya	0.44 (0.32, 0.60)	0.47 (0.27, 0.81)
Somali	0.29 (1.99, 0.41)	0.27 (0.14, 0.50)
Benishangul-Gumuz	0.34 (0.22, 0.53)	0.31 (0.15, 0.65)
SNNPR	0.91 (0.67, 1.25)	1.0 (0.58, 1.76)
Gambella	0.69 (0.34, 1.42)	0.66 (0.23, 1.91)
Harire	0.90 (0.38, 2.14)	0.88 (0.24, 3.23)
Tigray	0.35 (0.25, 0.49)	0.45 (0.25, 0.80)
Drie Dewa	0.84 (0.40, 1.76)	0.70 (0.24, 2.06)
Education		
No education	1.00	1.00
Primary	1.0 (0.96, 1.13)	0.95 ( 0.83, 1.09)
Secondary and above	1.4 (1.18, 1.76)	1.6 (1.02, 2.44)
Aware of community conversation program		
Not aware	1.00	1.00
Aware	0.80 (0.73, 0.87)	0.76 (0.67, 0.86)
Occupation		
Housewife	1.00	1.00
Working	0.74 (0.68, 0.79)	0.77 (0.69, 0.85)
Type of delivery		
Vaginal	1.00	1.00
Caesarian	0.73 (0.52, 1.01)	0.39 (0.22, 0.71)
Sex of newborn		
Male	1.00	1.00
Female	1.1 (1.04, 1.20)	1.2 (1.05, 1.30)

*COR* Crude Odds Ratio, *AOR* Adjusted Odds Ratio, 1.00 is the reference group

## Discussion

The Ethiopian infant and young child feeding guideline includes timely initiation of breastfeeding as one of the recommendations for newborn care [14]. Although timely breastfeeding initiation has been given due emphasis by the Ethiopian government, the optimal level of practice has not been reached. Significant variation of timely breastfeeding initiation was observed among Ethiopian regional states. Mothers who were from Amhara, Oromiya, Somali, Benshalgul-Gumz and Tigray regional states had lower odds of initiating breastfeeding within one hour as compared to mothers from Addis Ababa. This could be due to the fact that those mothers who reside in Addis Ababa have better access to health information and health facilities as compared to other regions.

Rural mothers had lower odds of timely breastfeeding initiation compared to their rural counterparts. A similar finding was reported from South Eastern Ethiopia which revealed that urban mothers had higher odds of timely breastfeeding initiation as compared to rural mothers [20]. This could be resulting from a lack of knowledge on the right time of breastfeeding initiation [21]. This might also be due to traditional beliefs and cultural practices such as colostrum discarding which are common among rural mothers in Ethiopia. Those mothers who discard the colostrum may take more than one hour to discard it and therefore initiate breastfeeding late.

Education was found to have paramount importance for timely initiation of breastfeeding. Similar findings were also reported by different studies [24–27]. Those educated mothers might be aware about the advantage of timely initiation of breastfeeding. They are also more likely to give birth in health facilities and to obtain the skilled assistance during delivery.

In this study, caesarian delivery was associated with delayed breastfeeding initiation compared to vaginal delivery. Caesarian delivery is the most consistent predictor of late initiation of breastfeeding reported by many studies conducted in Ethiopia and abroad [21, 25, 26] even though health facility delivery was associated with a higher rate of early initiation. During and immediately following emergency medical procedures such as caesarian delivery more attention may be given to the mother, while the infant feeding might be considered less important to address. Mothers might also be uncomfortable to breastfeed due to the pain experienced after surgery.

Delayed initiation of breastfeeding is commonly associated with malpractices such as prelacteal feeding and colostrum avoidance. Ensuring timely initiation of breastfeeding is a proximate indicator of colostrum feeding and avoidance of prelacteal food. Traditional beliefs, such as discarding colostrum, which are associated with delayed initiation of breastfeeding are common in rural societies of Ethiopia. This study also found the existence of gender bias in newborn care in Ethiopia. Female newborns had higher odds of experiencing timely breastfeeding initiation compared to male newborns. This may be due to traditional perceptions in Ethiopia that male infants privileged enough to receive prelacteal feeds are accepted by the society as strong and healthy.

Special attention should be given to educating those rural mothers regarding timely initiation of breastfeeding by the health extension workers and primary health care unit. It is expected that every mother gives birth in a health facility with a skilled provider to attend her delivery and provide newborn care. However, more than 75 % of Ethiopian mothers gave birth at home where newborn care is traditionally done by the mothers themselves. Therefore, to promote early initiation of breastfeeding in Ethiopia, women should be educated during pregnancy through existing health extension workers rather than waiting until the birth.

One of the strengths of this study is the use of national survey data. Therefore, the study findings can be used to inform policy and program actions. However, some administrative regional states had small sample sizes, which lessens the accuracy of prevalence estimates per region and should be interpreted with caution. Since this study is a secondary data analysis, other key variables such as traditional beliefs, post-birth activities and others are not included. This study also shares the limitation of a cross-sectional study design which makes it difficult to demonstrate cause and effect relationships.

## Conclusion

Timely initiation of breastfeeding was not optimal  in Ethiopia. Factors including place of residence, educational level, occupation, gender of the newborn and type of delivery should be considered in any intervention program to enhance timely initiation of breastfeeding among mothers in Ethiopia.
